# Great Lakes Resource at Risk

**DOI:** 10.1289/ehp.113-a164

**Published:** 2005-03

**Authors:** Scott Fields

For centuries the Great Lakes have been treated callously These five magnificent lakes—Superior, Michigan, Huron, Erie, and Ontario—located along the eastern half of the Canadian–U.S. border have served as a virtual sewer catching waste from industry, agriculture, commercial shipping, and households. Their natural barriers to other water systems have been breached, exposing indigenous ecosystems to aggressive invaders. They’ve been used as a highway for colossal ships that require deepened and broadened channels to crisscross the lakes, and that import exotic species along with their intended cargo. At times it could seem that this long-suffering water system will see no end of indignities. But recent renewed focus on the unique and tremendous value of this resource by governments and communities surrounding the lakes may turn the tide of neglect and abuse.

According to the U.S. Environmental Protection Agency (EPA), the Great Lakes contain 21% of the Earth’s and about 84% of United States’ surface freshwater. That’s about 22,000 cubic kilometers of water spread over 94,250 square miles. Each year the lakes provide more than 6.7 million cubic meters of water to municipalities and quadruple that to industry. They support a commercial fishery worth about $13 million as of 2002, according to the U.S. Geological Survey (USGS), and a sport-fishing industry of nearly $1.3 billion as of 2001, according to the U.S. Fish and Wildlife Service. Today about 25% of Canadians and 10% of Americans—a total of more than 33 million people—live in the Great Lakes watershed.

“The whole industrial expansion that took place during the ‘robber baron’ era [of the late nineteenth century] expanded along the shores of the Great Lakes,” says Deborah Swackhamer, a University of Minnesota professor of environmental chemistry. Soon ships were carrying iron ore, coal, and limestone from mines and quarries to steel mills and later steel to factories and products to markets. In addition to serving as a transportation system, the lakes provided a place to discharge manufacturing by-products.

Unlike a sewer, however, whatever enters this lake system stays awhile. On average, less than 1% of the five lakes’ water turns over each year, which means that many pollutants stay in place. They settle in sediments, adhere to other surfaces, become suspended in water, and bioaccumulate in organisms. Similarly, with the exception of migratory birds, most wildlife in the basin spend their entire life cycle in or near the lakes.

As a result of all these stressors, the lakes now house fish that are dangerous to eat, water that can be unsafe to drink, anoxic “dead zones”—areas in which virtually no plants or animals can survive—that appear each summer like clockwork, and an ever-growing population of unwanted species from other parts of the world.

## The Great Catch-alls?

According to the EPA, 362 contaminants have been identified in the Great Lakes system, only about a third of which have been evaluated for their effects on wildlife and human health. Two decades ago the International Joint Commission (IJC)—an organization that was formed by the Boundary Waters Treaty of 1909 to prevent and mediate boundary water disputes between Canada and the United States—identified 11 of these as “critical pollutants” that required immediate attention. The list includes polychlorinated biphenyls (PCBs), DDT, dieldrin, toxaphene, mirex, mercury, benzo[*a*]pyrene, hexachlorobenzene, furans, dioxins, and alkylated lead.

All of these substances bioaccumulate in organisms and persist in the environment. Joseph Makarewicz, a professor of environmental science and biology at The State University of New York at Brockport, explains that many of these chemicals are attracted to fats and repelled by water. “They readily move into tissue,” he says, “but basically it’s through phytoplankton, zooplankton, forage fish . . . into the various salmons and lake trout.” So the amount of contaminant in the water may be very small, even difficult to measure, but once it gets taken up by phytoplankton, it then biomagnifies—or becomes more concentrated—as it moves up through the food chain.

Over the past 20 years, as the American and Canadian governments have attempted to compel industries to better control or completely discontinue use of these substances, their levels in the environment have dropped. “We can demonstrate that DDT did definitely increase over the course of the fifties and sixties, peaked in the seventies, and then began to decline again,” Swackhamer says. “We can show the same for PCBs.” In response to the presence of these chemicals, over the years a smorgasbord of local, state, provincial, and federal public-awareness campaigns have been launched to alert citizens to the hazards of exposure to these chemicals.

The campaigns have met with mixed results. At-risk populations—those who consume large amounts of fish—are still being exposed, says Heraline E. Hicks, a senior environmental health scientist and manager of the Great Lakes Program at the Agency for Toxic Substances and Disease Registry. That’s because contamination with certain chemicals, such as PCBs, is ubiquitous in certain kinds of Great Lakes fish.

“People who are eating fish out of the Great Lakes as a major nutritional source are really getting hit hard,” says Keri Hornbuckle, a University of Iowa associate professor of environmental engineering. The problem, she says, is complex. Some groups of people, including low-income African Americans, Native Americans, and non–English speakers such as some Hmong immigrants, depend on the contaminated fish for their subsistence and can be hard to reach with or resistant to cautionary messages.

A suite of studies by different research groups suggest the health impacts may be profound. “We have seen changes in the sex ratio of children who were born to parents who were exposed to PCBs,” says Hicks. In a January 2002 *Journal of Occupational and Environmental Medicine* study of Lake Michigan fish eaters and their children, men with blood PCB levels of greater than 6 parts per billion were more likely to father male children than female children. The ratio of boys to girls in this population was about 154 boys for every 100 girls, whereas the normal human sex ratio is 103 boys to every 100 girls, says Hicks. Interestingly, a study in the 12 March 2003 issue of *Environmental Health* also found sex ratio effects, except that maternal exposure appeared to result in more girls.

Still another study, published in the February 1999 issue of *Environmental Research* showed that couples in which the man had a high body burden of PCBs due to his pattern of fish consumption took longer to conceive. And research published in the December 1997 *American Journal of Epidemiology* hinted at still more potential reproductive effects: women who ate PCB-contaminated freshwater fish experienced a shortened menstrual cycle.

Since the U.S. EPA banned production and most uses of PCBs almost three decades ago, levels of the chemicals in lake water have declined steadily. Over the years levels of PCBs—whose primary use was in nonflammable oil for such devices as switches and electrical transformers and capacitors, as well as a lubricant—have also dropped significantly in some species. According to the EPA, typical levels of PCBs in Great Lakes lake trout during the 1970s were 22 milligrams per kilogram. By the 1980s, they had dropped to about 6 milligrams per kilogram, just slightly above current levels.

Nonetheless, PCBs continue to cycle through the environment. They are mixed into sediments on the lakes’ floors, where they can be released by such disturbances as dredging or strong storms. Land sources continue to pollute as well, Hornbuckle says. The lightest of the 120 types of PCB compounds that have been found in the Great Lakes appear to remain active in the environment the longest.

And where do these long-banned compounds come from today? Hornbuckle has tracked them to industrial sites, many abandoned, surrounding the Great Lakes. Soils that were contaminated in the 1950s and 1960s in cities such as Chicago and Milwaukee appear to be an ongoing source of PCBs, she says. The chemicals volatilize on warm days, then enter the water system when blown over the lakes or the rivers and streams that feed them. Such atmospheric deposition is now the leading way PCBs are introduced into the lakes, Hornbuckle says. Plus, there are still PCBs remaining in service in older transformers and capacitors.

Mercury’s effects on human and environmental health are similar to those of PCBs. Today, says Michael Murray, a staff scientist with the National Wildlife Federation, much—if not most—of the mercury entering the Great Lakes comes via atmospheric deposition. Major sources of mercury releases to the air include coal-fired power plants, incineration of mercury-containing devices, mercury cell chloralkali plants, and industrial boilers.

From the 1940s through 1960s the lakes were bombarded directly with mercury-laden industrial waste. “Dow Chemical released about four hundred tons of mercury [into Lake Superior] from two chloralkali plants that they had,” says Michael Gilbertson, who recently retired as a biologist and secretary of the Workgroup on Ecosystem Health with the IJC. The result, he says, has been an outbreak among a series of communities on the Canadian side of the Great Lakes of suspected mercury-related poisoning. Symptoms observed have included tremors, deafness, and blindness.

In an unpublished dissertation, Gilbertson wrote that a review of Canadian hospitalization records reveals that in some communities near the lakes four times as many males are being admitted with cerebral palsy as in inland areas. Gilbertson says methylmercury is the only substance known to be associated with cerebral palsy, and males are more susceptible than females to neurological damage *in utero* from exposure to methylmercury.

More recently developed pollutants also threaten Great Lakes fauna—possibly including humans. Some that are appearing in the environment in increasing quantities are the polybrominated diphenyl ethers (PBDEs), which were introduced in the early 1970s as flame retardants in consumer products. “No one paid any attention to these compounds until the last couple of years,” says David Carpenter, a professor of environmental health and toxicology at The State University of New York at Albany’s Institute for Health and the Environment, “and now we’re just finding them everywhere.”

Although PBDEs are mixed with plastic polymers during manufacturing, they don’t bind chemically with the plastic. As a result, they leach readily from products that contain them. Like PCBs, PBDEs concentrate in fats. That and their resistance to degradation combine to create a persistent chemical that bioaccumulates. “Their manufacture increased exponentially over the last decade or so, and we’re seeing them in fish and in sediments with that same increase,” Swackhamer says. “We can go back to the sediments since we didn’t measure them in the environment until a few years ago; we can actually use the sediments to go back and demonstrate the fact that they’ve increased exponentially all through the mid-eighties and into the nineties.”

PBDEs have now been found in fish from all five of the lakes. According to a University of Wisconsin study published in the 1 March 2001 issue of *Environmental Science and Technology*, Lake Michigan salmon (which are an introduced species managed for sport fisheries and sustained through stocking programs) contain PBDEs at levels above 100 parts per billion, one of the world’s highest concentrations for salmon in open waters. The authors also found PBDEs in some forage fish, such as alewife and smelt. Levels of PBDEs also appear to be increasing in human tissue, according to a review published in the May 2000 issue of *EHP*. Although the human health impacts of PBDEs aren’t well understood, these chemicals have been shown in animal studies to have effects similar to those of PCBs, including effects on brain development, learning, memory, thyroid levels, and reproduction.

The sources of PBDEs are ubiquitous. “PBDEs are used everywhere—in furniture, carpeting, computers, and vehicles,” says Sergei Chernyak, a research scientist in the University of Michigan School of Public Health. To gauge the contribution of domestic sources of PBDEs to the environment, Chernyak’s research team compared PBDE levels inside and outside homes. The levels inside a typical house were 70 times the levels just outside the building. “Although this was a pilot study, it seems that houses have strong sources of these contaminants, which is a public health concern,” Chernyak says.

With such a diffuse source, stemming the flow of PBDEs to the Great Lakes poses a much more difficult problem than that of PCBs, whose original sources could be traced to large industrial sites, Chernyak says. “The source to the lake came not from water nor from the shore but probably from [the air],” he says.

Such pollutants can devastate wildlife in subtle and hard-to-detect ways, says Philip Cook, a research chemist for the U.S. EPA. For example, research by Cook and colleagues published in the 1 September 2003 issue of *Environmental Science and Technology* indicates that the introduction of dioxins and certain related PCB compounds to Lake Ontario in the 1950s spelled the end of lake trout, the lake’s top predator. “This was a finding that would contradict prevailing opinions in the Great Lakes, specifically that lake trout, which had declined to extirpation—in other words the population was gone by 1960—was due to other ecological factors, not chemical toxicity,” Cook says. Exotic species, overfishing, loss of habitat, and water quality reductions had all been blamed, he says, but “we think there is evidence to show that at least up until very recently this toxicity was a factor that impaired any success in trying to reintroduce the species.”

Sewage poses still another threat to the lakes. According to the IJC’s September 2004 *Twelfth Biennial Report on Great Lakes Water Quality*, each year rainstorms send trillions of gallons of untreated human sewage into the lakes. In 2001, Lake Michigan alone absorbed 52 billion gallons of sewage and partially treated wastewater.

With the sewage come nitrates, which can cause gastric problems and methemo-globinemia if they find their way into drinking water in excessive amounts. It also brings any number of pathogens, including the *Cryptosporidium* parasite and *Escherichia coli*, which can cause severe, and sometimes deadly, intestinal diseases.

Today most raw sewage contributions to the Great Lakes are inadvertent, and they most often come from overflowing sewer systems during storms. A smaller, but still significant, source of sewage is the thousands of privately owned septic systems on the farms, vacation homes, and other rural dwellings that ring the lakes, says Kathleen Halvorsen, an associate professor of natural resource policy at Michigan Technological University. Some 10–20% of these systems are failing, she says. Still, she adds, it’s the big cities that contribute the most untreated sewage.

## The Great Melting Pots?

Almost as diverse as the pollutants that have found their ways into the Great Lakes are the plants and animals that now call the lakes home. The Great Lakes connect to the Atlantic through the Saint Lawrence River, which flows out of Lake Ontario. But until the early 1800s Niagara Falls, at the west end of Lake Ontario, prevented water and the animals that live in it from reaching the rest of the Great Lakes system. In the 1800s, however, two waterways—the Erie Canal and the Welland Canal—opened these lakes to the Atlantic Ocean. These new channels provided a foot in the door for invasive species, some traveling under their own power, others hitching rides aboard the ships that began plying the waters.

Since the early 1900s, 170 or so nonnative species have found their way into the Great Lakes, says Edward Mills, a fish ecology professor at the Cornell Biological Field Station. Some, such as the zebra mussel, stowed away on oceangoing ships. Some, such as the rusty crayfish, escaped their fate as bait for anglers. Some, such as the banded mystery snail, were liberated from home aquariums. Some, such as garden loosestrife, were cultivated. Some, such as the orange-spotted sunfish, swam in through man-made canals. And some, such as the rainbow trout, were introduced intentionally as game fish.

These newcomers can devastate their new homes. By 1947, for example, the sea lamprey—an eel-like fish that sucks bodily fluids from other fish—had invaded all of the lakes. The lamprey—which probably swam up the Hudson—has contributed to the collapse of whitefish and lake trout fisheries. A more recent and possibly more damaging invader, the zebra mussel, arrived in the ballast water of a transoceanic vessel. Since its first Great Lakes sighting in 1988, this fingernail-sized mollusk has found its way into seemingly every nook and cranny of the lake system.

Zebra mussels and their fellow invaders, quagga mussels, are the only mollusks that can attach themselves firmly to solid objects. The solid objects they select for their colonies include cooling pipes at power plants, boat hulls, propellers, and docks, which—at mussel densities of up to 70,000 per square meter—quickly become clogged and fouled. They’re also radically altering the ecological balance of the lakes, Makarewicz says. The mussels are such effective filter feeders that they strip water of the various plankton that indigenous creatures eat. In the process their fatty tissues accumulate concentrations of PCBs, methylmercury, and other contaminants at about 10 times the density of native mussels, giving the fish and waterfowl that feed on them an extra shot of biomagnified poison.

In not much more than a decade, Makarewicz says, the zebra and quagga mussels have undone years of work and at least $5 billion spent on Lake Erie alone to upgrade water quality by reducing phosphates from fertilizers, detergents, and industrial discharges. “There was major improvement in all of the basins of Erie,” he says. “The walleye populations that [fisheries] began stocking—which is a sport fish—came back in big numbers and things looked very good. And then the zebra mussels changed that.” Although phosphates are largely under control, he says, invasive mussles have re-degraded the quality of the water by overclarifying it.

Zebra mussels, quagga mussels, and a fish known as the round goby have been tagged as the most likely culprits in recent type E botulism outbreaks in Lake Erie. “The real mechanism has not been clearly distinguished yet,” Mills says, “but it does look like it’s some relationship between the mussels—mostly quagga mussels—and gobies.” The highly efficient mussels clarify the water so much that sunlight can reach normally shaded aquatic plants and promote their growth. When the unwonted proliferation of growth dies, its decay consumes oxygen, providing ideal conditions for the *Clostridium botulinum* bacteria to grow. The mussels also may concentrate *C. botulinum*, which is biomagnified when the mussels become meals for round goby. “We know gobies feed on mussels,” Mills says. “The gobies then get the *Clostridium* bacteria toxin. They die and then whatever eats them—it could be fish or birds feeding on them as they roll in on shore—then gets the toxin and dies.”

Similarly, says R. Peter Richards, a water quality hydrologist at Ohio’s Heidelberg College, zebra mussels may be the cause of recent increases in blooms of the toxic algae *Microcystis*. “It is at least a nuisance algae, if not a potential health problem,” he says. “The zebra mussels don’t like to eat *Microcystis*, so they suck all of the particles out of the water and they spit the *Microcystis* back out. At the same time they digest all the other stuff and spit it out as pseudofeces, which releases nutrients that feed the *Microcystis*. Zebra mussels are killing off the competition and fertilizing the *Microcystis*.”

In spite of a 1993 rule that calls for oceangoing ships to flush their ballast tanks before entering the Great Lakes system, the influx of invasive species has actually increased. One reason may lie in the loophole that ships that are fully loaded with cargo, and so in theory are free of ballast water, aren’t required to flush their tanks. But even “empty” ballast tanks can contain up to 10 tons of sediment and trapped water, and species can be released into the lakes as the ships unload and reload cargo.

Another reason may be the types of species that are coming in. Spiny water flea spores have been identified in ballast-tank residue, and it’s possible this is how this and other alien species have found their way into the lakes. In inhospital conditions, mating spiny water fleas produce “resting eggs” that are first carried in the females’ brood pouches and later released to sink to lake sediments, where they lie dormant until the water warms in spring or summer. The creature’s sharp spines make it impossible for all but the largest predators to eat them. Meanwhile, the spiny water flea itself competes aggressively for the plankton that so many native species depend on for food.

Flushing ballast, even if made more effective, won’t control invaders that travel to the lakes through other routes, such as the Asian carp, which is knocking on the lake system’s southernmost door. In an attempt to stop the voracious fish, an electrified barrier has been strung across the Chicago River, which flows out of Lake Michigan. So far Asian carp—a term used for multiple species of carp used in Southern aquaculture—have not been found in the Great Lakes. But they are less than 50 miles away on the Illinois River, where in some stretches they have become the dominant species.

Asian carp, which include bighead carp and silver carp, are thought to have escaped from U.S. fish farms during the Mississippi River floods more than 10 years ago. They are used for pet food because they quickly grow quite large. Bighead carp can weigh more than 100 pounds and because they can eat as much as 40 pounds of plankton per day, some ecologists warn that they could equal or even outdo zebra mussels in terms of how efficiently and quickly they ravage the ecosystems they invade.

In threatening native species, these invaders also reduce biodiversity, says Dora Passino-Reader, a USGS fishery research biologist. Zebra mussels, for example, reproduce so quickly, are so hardy, and (for a mussel) are so aggressive—as many as 10,000 have been found affixed to the shell of one native mussel—that they suppress native mussels’ movement, feeding, and reproductive behavior. To take another example, several species of cisco, which were already depressed by the mid twentieth century by overfishing, were then dealt the final blow by three exotic species—the parasitic sea lamprey, a predator, and the alewife and rainbow smelt, competitors. “Some of these species have disappeared,” says Passino-Reader. “[Ciscoes are] not located in the Great Lakes at all anymore. They aren’t able to live in the Great Lakes after the invasion of the alewife. The competition and predators have changed so much that these species have actually been lost.” Alewife are also high in the enzyme thiaminase, she says, which breaks down vitamin B_1_ (thiamine) and contributes to mortality in the offspring of the alewife’s predators.

Although programs to eliminate sources of known pollutants—notably PCBs, DDT, and mercury—have largely stemmed the flow of these substances into the lakes, foreign species have proven more stubborn, Mills says. “When these species get in, it’s pretty much irreversible,” he says. “From the chemical side of things, once you control a nasty chemical you have some hope that the system will respond, and usually it responds quite quickly. But on the biological side of things, very rarely does an invasive species go extinct. The system has to change very dramatically for that to happen.”

Invasive species are not the only cause of the Great Lakes’ biodiversity woes, however. According to Lucinda Johnson, associate director of the University of Minnesota’s Center for Water and the Environment, the Great Lakes system’s coastal marshes, sand dunes, rocky shorelines, prairies, savannas, and forests—and the creatures that live in them—are being displaced by human development. For example, coastal marshes, which house an estimated 30% of Great Lakes species, are also a frequent victim of development itself as well as the fallout from development: toxic runoff rich in road salt and sediment. As the large cities that ring the Great Lakes continue to grow, sprawl fallout such as subdivisions, roads, golf courses, and vacation homes poses an ever-increasing threat to Great Lakes ecosystems.

## The Great Warm-up?

Recent weather trends over the Great Lakes have included decreased precipitation, higher-than-usual air temperatures, and less ice cover in winter. As a result of these influences, water levels in Lake Michigan and Lake Huron dropped faster from 1998 to 2002 than during any other recorded period, according to research from the National Oceanic and Atmospheric Administration (NOAA) published in the August 2004 *Bulletin of the American Meteorological Society*. In recent years Lake Superior has been at its lowest level since 1926, Lake Erie hasn’t been as low since 1966, and Lakes Michigan and Huron are at their lowest levels since 1965.

Some environmentalists have pointed to these alarmingly low water levels as evidence of global warming. But, says Douglas Wilcox, branch chief for coastal and wet-land ecology at the USGS Great Lakes Science Center, the current low levels are still within the realm of low lake levels on the upper lakes that have been seen through recorded and even prerecorded history. Wilcox says the long-term data suggest we could be entering a longer-term warming cycle with a naturally resulting long-term low lake level cycle. “On the other hand,” he says, “if you throw in anthropogenic warming on top of that natural cycle, maybe the low lake level that will occur seventy-five or a hundred years from now—when the low cycle occurs again—may be much lower than it would have been naturally if it had-n’t been for human impacts.”

Low lake levels are necessary, Wilcox explains, for natural restoration of lake wetland ecosystems. During low water levels, sediments are exposed allowing other lake plants to germinate and grow. The resulting wetlands provide habitat for species such as frogs and wading birds. But for people who depend on the lakes for their livelihood, lower water means lower profits. In 2000, for example, lake carriers, which move such cargo as ore, coal, and grain within the lake system, had to reduce their loads by 5–8%, according to a 2004 NOAA brochure titled “Water Levels of the Great Lakes.” Sustained low water periods invariably bring with them political pressure to deepen shipping channels and harbors, says Emily Green, the Sierra Club’s Great Lakes program director. But dredging in these lakes can mean releasing pollutants that have settled into lake bed sediments.

Hornbuckle says the Great Lakes’ infamous violent storms can resuspend PCBs and DDT compounds that have settled into sediments. And with climate change, Johnson says, have come more of the violent storms, and storms that are more violent than before. “There has been a doubling over the last century in the number and intensity of storm events, and those [trends] are expected to hold into the next century,” she says. “The prediction is that there will be an additional doubling of intense storm events.” The effects of these powerful storms, she says, will be magnified by increasing human development—with its destruction of wetlands and addition of impervious surfaces, such as roads and sidewalks—in the lakes’ watershed. This, she says, is a recipe for increased lakeshore erosion, water turbidity, and resuspension of sediments.

R. Michael McKay, a biology professor at Bowling Green State University, says plummeting water levels would also worsen another ongoing problem: dead zones. Lake Erie, for example, has periodically suffered bouts of anoxia. Naturally occurring ridges, or “sills,” divide the lake into three basins. Many summers nearly all of the dissolved oxygen in the central basin is consumed, dooming those creatures that cannot escape to the west or east. “Because of thermal stratification in summer there is no way of getting new oxygen down into the bottom water,” McKay says.

## A Great Future?

A number of programs are under way to address problems in the Great Lakes [for in-depth coverage of these programs, see “The Great Lakes: Awash in Policy,” p. A174 this issue]. Perhaps some stressors on the Great Lakes can be mitigated, but others are here for the long haul, Cook says. “Once you pollute the Great Lakes it takes a long, long time for these very persistent chemicals to clear from the system so that levels can return to presumably noneffect levels,” he says.

## Figures and Tables

**Figure:**
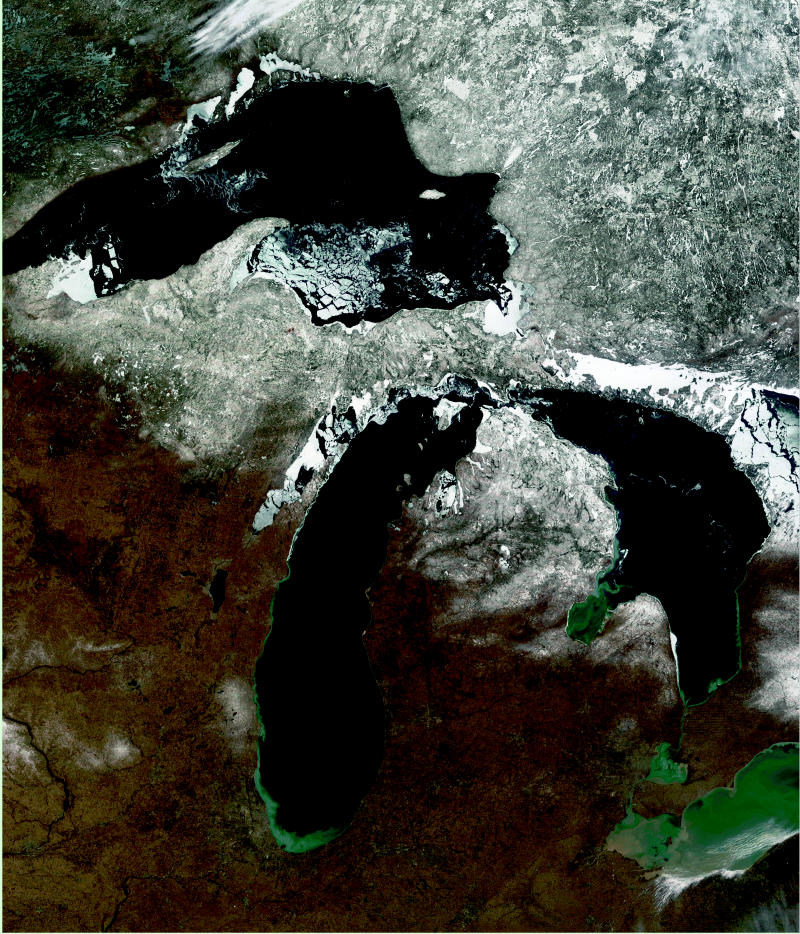


**Figure:**
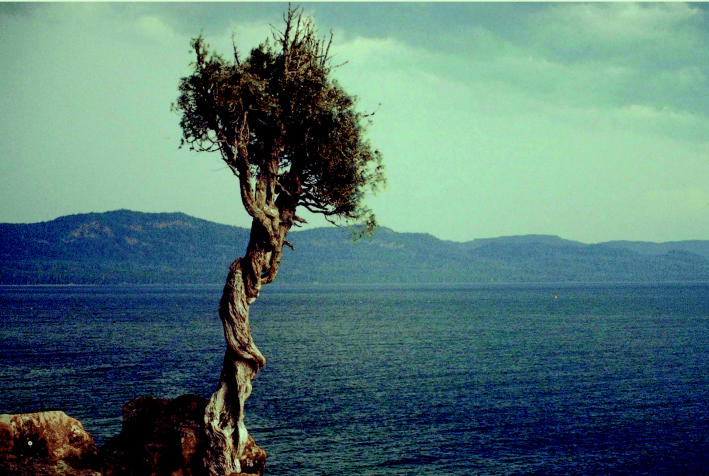
**Natural treasures.** The Great Lakes (seen here from the vantage point of the Witch Tree at Lake Superior Grand Portage in Minnesota) are a priceless natural resource, but decades of abuse and pollution are taking their toll on these once pristine bodies of water.

**Figure:**
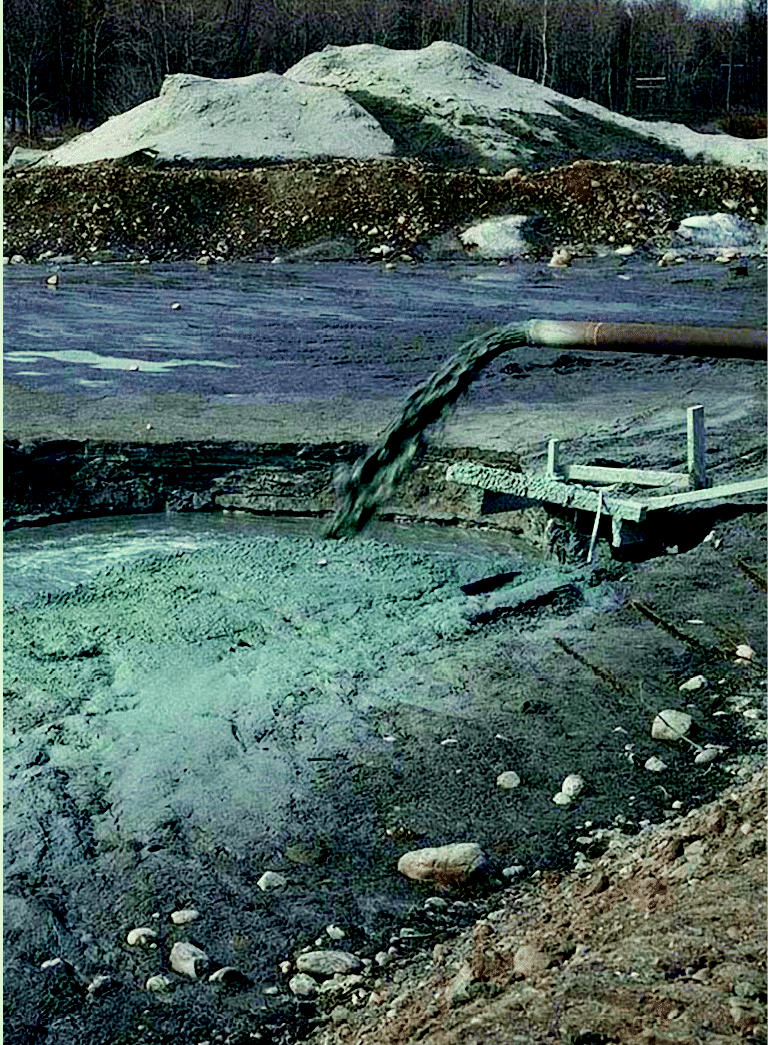

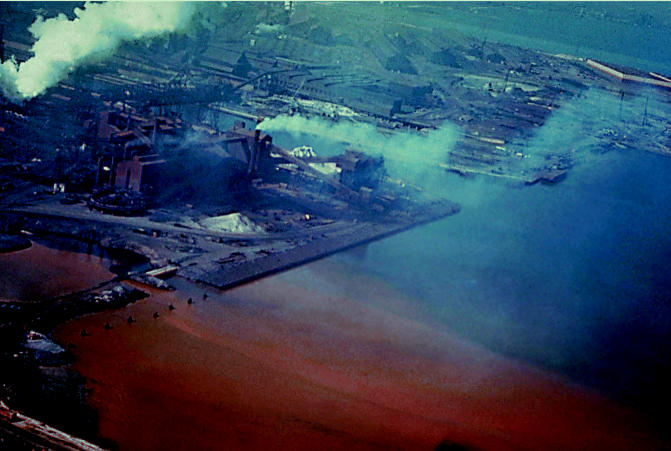
**Industrial waste bins.** Industries along the lakes including paper companies, power plants, chemical manufacturing, and myriad others have routinely polluted the waters and ecosystems.

**Figure:**
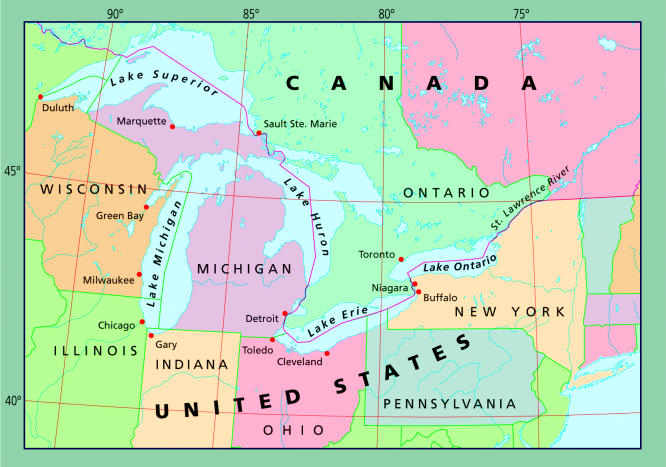


**Figure:**
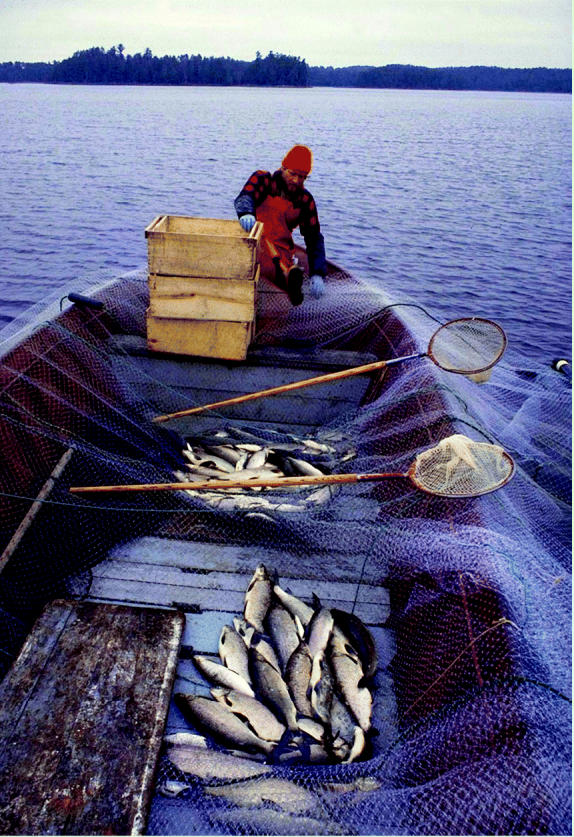

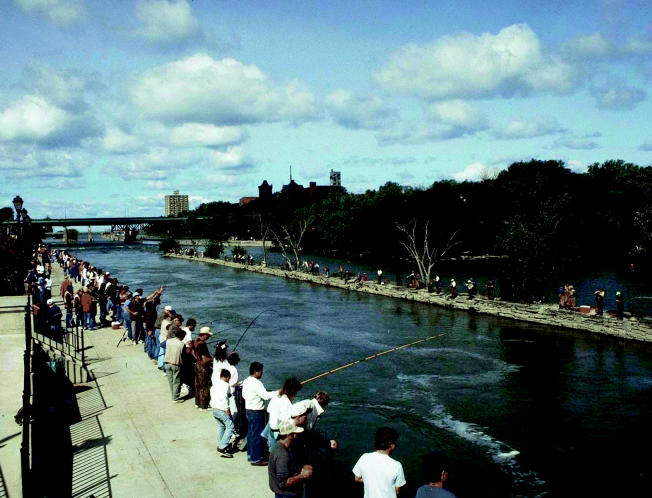
**Failing the fish, the fishermen, and the folk alike.** Pollutants are accumulating in Great Lakes fish, making many of them unsafe to eat. This hazard threatens the livelihoods of many and the health of even more. (Above: trap net whitefish in Lake Saganaga Bounding Waters Canoe Area Wilderness, Minnesota; left: early fall fishing on the Oswego River, Oswego, New York.)

**Figure:**
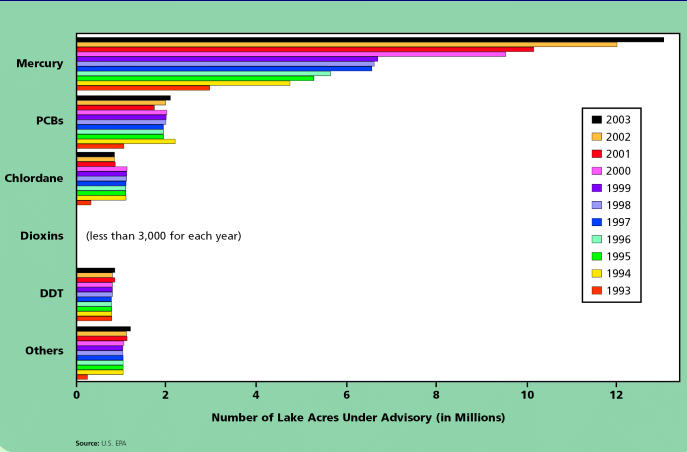
**Fish Consumption Advisories:** Acres of U.S. Lakes Under Advisory for Various Pollutants

**Figure:**
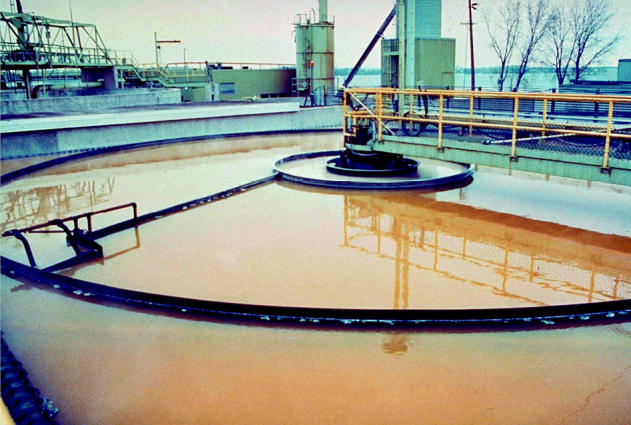

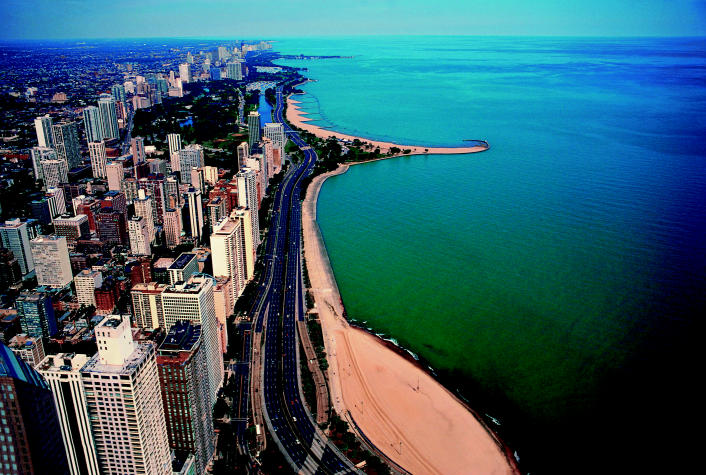
**Teeming at the water’s edge.** Ever-encroaching cities such as Chicago, Illinois, draw on Great Lakes resources while dispensing sewage and refuse into the waters at alarming rates—and with escalating consequences.

**Figure:**
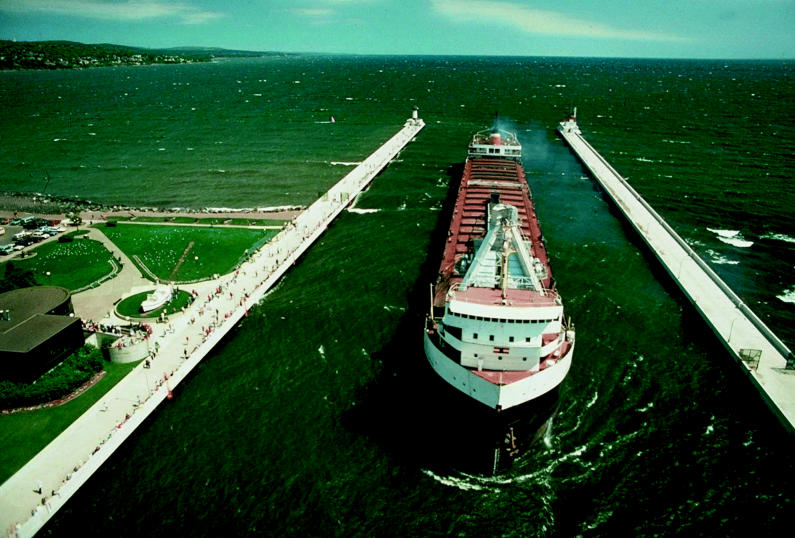

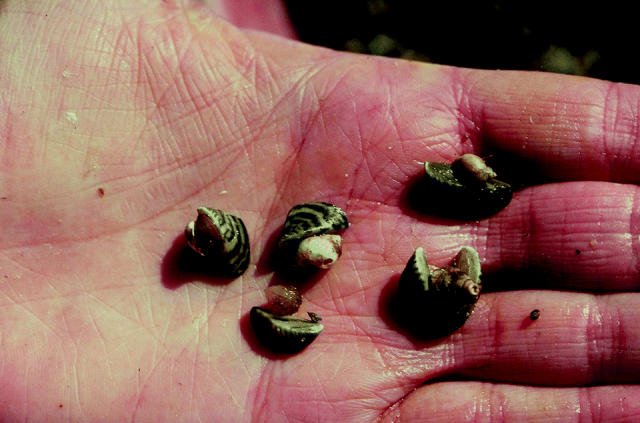
**Ships of fate?** A ship arrives in Duluth ship canal in Minnesota (above), just one of thousands that ply the Great Lakes every year. Along with the myriad goods in their hulls, their ballasts carry invasive species such as zebra mussels (right), which can have disastrous effects on infrastructure and native ecosystems.

**Figure:**
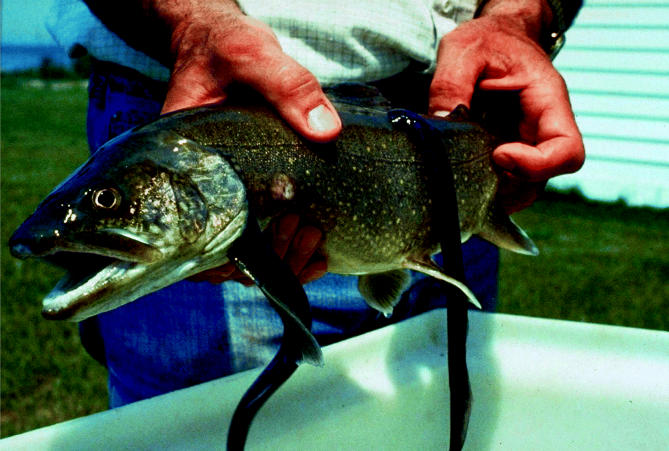

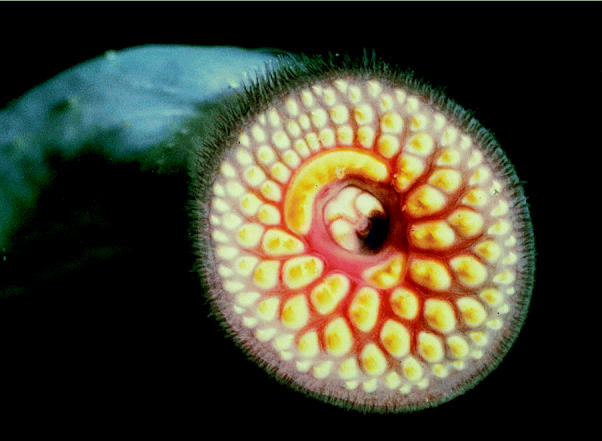
**A sticky situation.** The sea lamprey (right), a nonnative species that probably swam into the Great Lakes from the Hudson River, attaches to lake trout and whitefish (above). Sea lampreys have invaded all the Great Lakes and wiped out fisheries.

**Figure:**
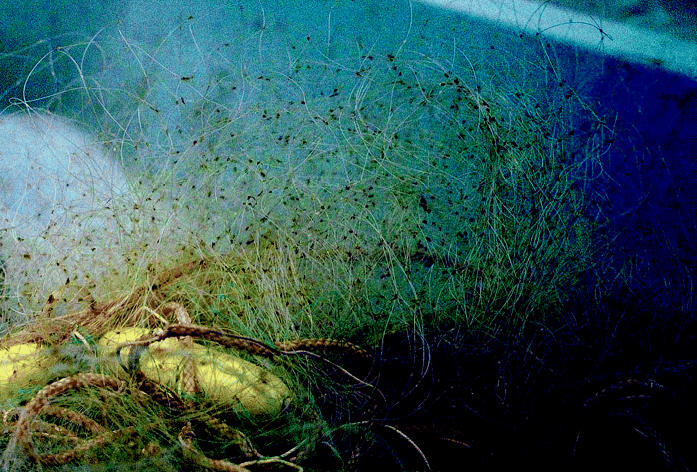

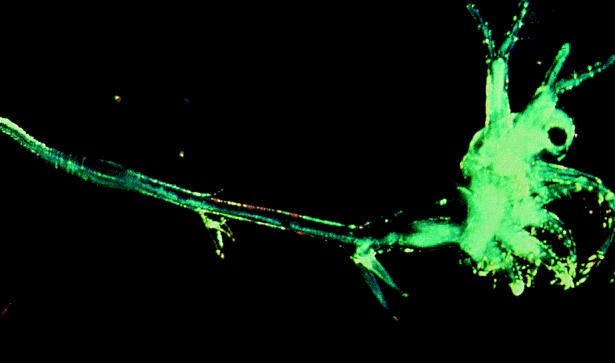
**Tiny hitchhikers.** Spiny water fleas (above in a gill net; close up at right) are one of many new invasive species that arrive as dormant eggs in ship ballast and later hatch in the lakes.

